# Early detection of parenting stress in mothers of preterm infants during their first-year home

**DOI:** 10.1186/s40359-020-00435-z

**Published:** 2020-06-23

**Authors:** C. Lau, M. R. Turcich, E. O. Smith

**Affiliations:** Department of Pediatrics, Baylor College of Medicine, Houston, TX 77030 USA

**Keywords:** Parenting[al] Stress Index [stress index], Preterm mother-infant dyad, Maternal stress perception

## Abstract

**Background:**

Maternal stress following the birth of an infant is well acknowledged. It is particularly so when infants are born prematurely as their mothers cannot fully take on their parenting role until their infant(s) is discharged from neonatal intensive care units (NICUs). In this exploratory study, we examined whether these mothers’ parenting stress would lessen during their first-year reunification with their infant(s) as they settle into motherhood at home.

**Methods:**

Two groups of mothers with infants born between 24- and 33-week gestational age were recruited. A group of 25 mothers were monitored at their infants’ 1-month corrected age (CA) and a second group of 24 mothers were monitored at their infants’ 12-month CA. Subjects completed the long form Parental Stress Index (PSI) ranking how stressful they *perceive* the individual subscales in the Child and Parent Domains of the self-reported questionnaire (PSI-3; Abidin; PAR Inc). The PSI theorizes that the stress mothers *perceive* is a resultant of their respective characteristics, interactions with their infant(s), family, and environment. Statistical analyses include descriptive statistics, χ^2^ square analysis, and independent t-test.

**Results:**

There was no significant difference in the levels of *perceived* stress in the PSI subscales between the two groups of mothers at 1- and 12-month CA. Scores for the majority of respondents fell within the 15th to 80th percentile (% ile) distribution of Abidin’s normative population, with some mothers falling below the 15th % ile.

**Discussion/conclusion:**

The data collected suggest that: 1. the perceived stress experienced by mothers during their first-year reunited with their preterm infants is within the normal range observed in Abidin’s normative population. 2. As the PSI is a self-reported survey, care providers need to be aware that some mothers may downplay their stress responses. 3. With the ability to monitor *individua*l participants, the PSI can be readily offered to mothers at their infants’ first year routine clinical visits to assist in the early identification of parenting issues that may threaten the development of a healthy mother-infant dyad. Early appropriate guidance and social support would help “at-risk” mothers develop more constructive parenting routines.

## Background

Human infants, due to their immaturity at birth, must rely on their mother/caregiver to survive [1]. If mother and fetus are viewed as an inseparable entity during their intra-uterine development, mother-newborn during infancy may be regarded in a similar fashion. The continuous interfacing between a mother and her child(ren) will determine the nature of their bond/attachment [2, 3]. It is a complex psycho-physiologic process that first and foremost depends on the proper initiation of maternal behavior/motherhood [4]. The latter is dependent upon specific maternal genetic, neuroendocrine, immune, and behavioral alterations occurring in response to her sustained contact with her child [4–11]. At the same time, appropriate infant responses towards the parent is germane for the maintenance of healthy dyadic exchanges [10, 12–14]. Indeed, one would envision that healthy mother-infant exchanges would be *mutualistic* with a balanced “give and take” as illustrated by a smooth behavioral synchrony and the development of positive attachment between mother and child [15–18]. If detrimental, unbalanced exchanges, such as those resulting from maternal/infant ill-health, stressful environment, family imbalances, would lead to unfavorable consequences straining their relationship and likely increasing the stress level of both partners of the dyad [14, 17, 19–21]. With stress encompassing physical, mental, and physiologic elements interfering with an individual’s normal activities, one can appreciate how stressors can readily challenge the balanced exchanges between a mother and her infant(s). This is particularly germane to mothers who deliver prematurely as the immaturity and fragility of their high-risk infants often require a period of hospitalization in a neonatal intensive care unit (NICU) and forced separation of mother and child. The latter puts them at greater risk of developing suboptimal relationships when compared with their counterparts who deliver healthy term infants. The literature relating to maternal distress, anxiety, depression among mothers of preterm infants has grown as survival of these infants is increasing [17, 21–29]. Interventions have been proposed to assist mothers’ behavioral sensitivity towards specific issues encountered by their children, e.g. cognitive/language development, behavioral problems [30–32]. However limited attention has been given to “early” identification of how a mother’s *perception of* stress due to particular characteristics of her child may threaten the development of a stable mutualistic parent-child system over the long term (1, 13,16). Consequently, this exploratory study was initiated based on the *working premise* that mothers may be “viewed” as the partners who have greater pro-active influences over their dyads’ interactions due their essential parenting responsibilities [33]. As such, its objective was to assemble descriptive and normative parenting stress information on the early development of mother-preterm infant systems post-NICU discharge using the long form of Abidin’s Parenting Stress Index (PSI-3, PAR Inc) when their infants reached 1- and 12-month corrected age (CA). It was reasoned that mothers’ *perceived* stress when interacting with their infants would be greater at 1-month CA than 12-month CA as mother and infant settle into their respective routine interactions.

## Methods

This study included a convenient cross-sectional sample of two groups of 25 and 24 mothers monitored at their preterm infants’ 1-month and 12-month CA, respectively. Infants were born between December 2005 and March 2009 between 24 and 33 weeks gestational age. The participants were part of a larger prospective study conducted between August 2005 and July 2011 at the General Clinical Research Center (GCRC) of Texas Children’s Hospital/Baylor College of Medicine (Houston TX, USA) that investigated the benefits of various oral feeding interventions on infants’ feeding/eating behaviors and maternal stress through their first 2 years home [14, 17, 34, 35]. None of these infants were diagnosed with hydrocephalus, intraventricular hemorrhage grade III and IV, necrotizing colitis, bronchopulmonary dysplasia, or congenital anomalies. The study was approved by the Baylor College of Medicine Institutional Review Board for Human Research. Written informed consent was obtained from mothers. Following infants’ discharge from the NICU, mothers were to return to the GCRC at Texas Children’s Hospital for their infants follow-up visits and their own psychological assessments.

The PSI is a clinical tool developed to assess the multidimensional factors that may affect a mother-child dyad (PSI-3, PAR Inc). Its validation and reliability have been substantiated. It is recommended as a *screening and interpretive tool* for evaluating the state of the parenting system as it is *perceived* by the parent. Its aim is to offer *interpretive* guidelines to help identify issues that may lead to problems in the child’s or parent’s behavior that may aggravate parenting. As such, PSI scores are not to be used as a diagnostic tool, but rather as working hypotheses that health professionals may use to assist individual parents.

**Fig. 1:**
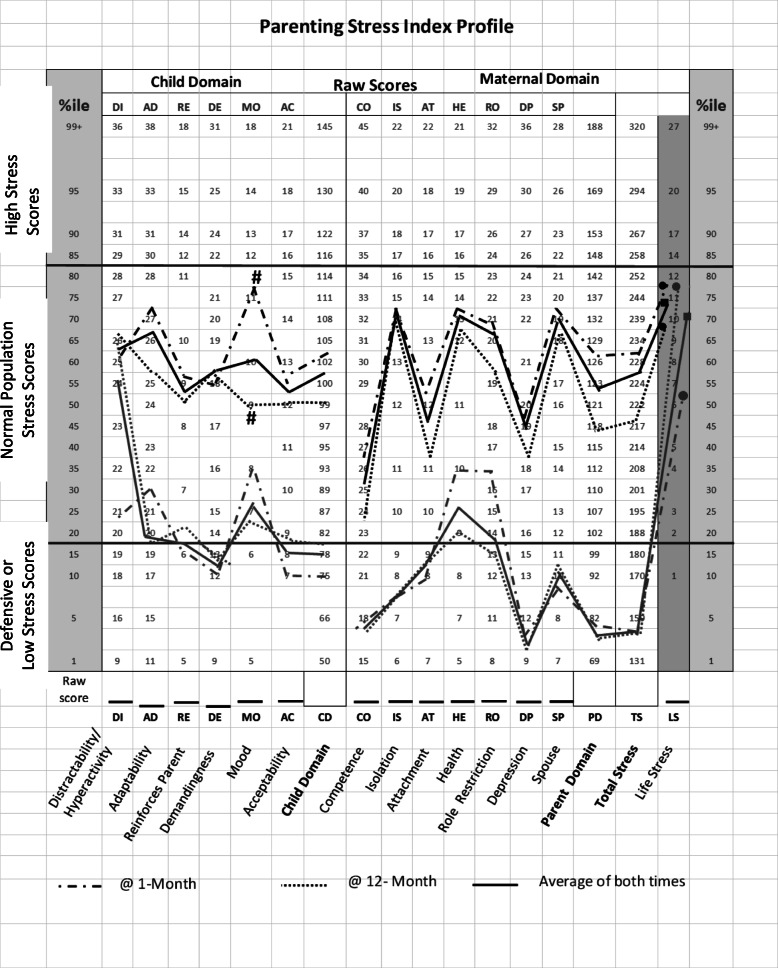
PSI profiles of Non-Defensive (ND; black lines) and Defensive (D; grey lines) mothers at 1-month CA (dashed line), 12-month CA (dotted line) and average of both times (solid line); %ile columns: frequency distribution of Abidin’s normative population; Maternal Raw Scores in Child and Maternal Domains; Y-axis: subscales in Child and Maternal Domains; Child and Parent Domain (CD; PD) Total Stress (TS); Life Stress (LS)

The instrument consists of a Child Domain with 6 subscales (Distractibility/Hyperactivity, Adaptability, Reinforces Parent, Demandingness, Mood, and Acceptability) and a Parent Domain with 7 subscales (Competence, Isolation, Attachment, Health, Role Restriction, Depression, and Spouse/Parenting partner relationship) [Table 1; Fig. 1].

**Table 1:** Parenting[al] Stress Index (PSI) subscales

**Maternal Stress Perceptions of Child Domain Subscales**	
Distractibility/ Hyperactivity (DI)	High raw scores associated with behaviors, e.g., overactivity, restlessness, distractibility, short attention span, does not seem to listen, or Attention Deficit Disorder with Hyperactivity.
Adaptability (AD)	High raw scores associated with characteristics that make parenting task more difficult due to child’s inability to adjust to changes in physical or social environment
Reinforces Parent (RE)	High raw score associated with parents not experiencing positive reinforcement from child
Demandingness (DE)	High raw scores associated with parent experiencing child as placing many demands
Mood (MO)	High raw scores associated with child whose affective functioning shows evidence of dysfunction
Acceptability (AC)	High raw scores associated with child’s physical, intellectual and emotional characteristics that do not match parental expectations
Child Domain (CD)	Total stress raw score of above Child Subscales
**Maternal Stress Perceptions of her Own Parental Domain Subscales**	
Competence (CO)	High raw scores may be due to factors such as parent’s real or perceived inability to care for child, lack of acceptance/criticism from spouse, first time parent’s inexperience, limited child management skills
Isolation (IS)	High raw scores may be associated with social isolation from peers, relatives due to child care
Attachment (AT)	High raw scores may be associated with absence of emotional closeness with child, e.g., cold pattern of parent-child interactions, parent’s real or perceived inability to understand child’s feelings and/or needs accurately
Health (HE)	High raw scores may suggest deterioration in parental health
Role Restriction (RO)	High raw scores suggest parental role as restricting parent’s freedom and frustration in attempting to maintain his/her own identity
Depression (DP)	High raw scores are suggestive of significant parental depression
Spouse (SP)	High raw scores relate to parents who lack the emotional and active support of the other partner in child management
Parental Domain (PD)	Total Stress raw scores of above parental Subscales
Total Stress (TS)	Sum of Child and Parental Subscales (CD + PD)
Life Stress (LS)	High raw scores relate to current stress experienced outside the parent-child relationships

The PSI was used to monitor two interpretive measures. First, the percentile scores of subjects’ raw scores were derived from the frequency distribution of Abidin’s normative population that included 2633 mother/child between 1 month and 12 years of age recruited primarily in pediatric clinics and schools (%ile; 1–99%; Fig. 1). Respondents’ %ile scores falling within the 15th and 80th %ile (demarcation lines, Fig. 1) are deemed within Abidin’s normative population range. Scores ≥85%ile are indicative of clinically significant high stress, while %ile scores < 15% may be indicative of low stress or respondents’ “Defensive Responding” attitude. Second, the PSI Defensive Responding scores are used as a measure of the truthfulness of participants’ answers. With Defensive Responding scores > 24, participants are deemed truthful or “Not Defensive (ND)” in their responses. With scores ≤24, participants are deemed “Defensive (D)” and care is recommended when interpreting their responses as they may be downplaying their stress or being truthful due to their high competency in infant caring and/or supportive environment.

As it is recognized that individuals’ responses to self-reported psychological questionnaires may be downplayed as a function of their ‘social desirability’ attribute, i.e., the need for social approval or the avoidance of social disapproval, the Marlowe-Crowne Social Desirability test was also administered to all mothers to obtain another independent measure of participants’ response bias/truthfulness. With this instrument, the higher the score, the more likely a subject’s response is reflective of their social desirability trait rather than their truthfulness [36–38].

Statistical analyses include descriptive statistics, χ^2^ square analysis, WINPEPI (Compare2), and independent t-test with significance attained at *p* ≤ 0.05. χ^2^ square analysis for categorical variables and WINPEPI(Compare 2) were used to identify potential differences in the stress scores of the Child and Parent Domains subscales at 1- vs. 12-month CA. Independent t-test was used to determine whether individual maternal PSI subscales scores differ between ND vs. D groups and within group of mothers at 1- vs. 12-month CA, respectively. It was assumed that with each subscale being independent from each other, maternal self-reporting is solely a function of the mothers’ individual disposition and/or functions/dysfunctions.

## Results

Participants’ characteristics are presented in Table 2. Twenty-five mothers were monitored at the 1-month CA visit and 24 others at their infants’ 12-month CA visit. In the case of multiples births, PSI was only monitored for maternal responses to one infant. Twelve mothers at the 1-month CA visit delivered multiples (12/25) vs. seven at the 12-month CA visit (7/24). No difference was observed in characteristics between the 1- and 12-month CA groups of mothers (Table 2).

**Table 2:** Subjects Characteristics

Maternal- & Infant Demographics	Mothers @ infant 1-month post NICU discharge	Mothers @ infant 12-month (Corrected Age)	*P* value
n	25	24	
**Multiples (%)**	12 (48%)	7 (29%)	ns^¥^
**Maternal Age**^**a**^	31.36 ± 7.13	32.08 ± 5.52	ns^#^
**Defensive Responding (n)**	2	5	
**Gestational Age (weeks)**^**a**^ (range)	28.65 ± 2.45 (24.3–32.9)	28.30 ± 1.95 (24.3–32.3)	ns^#^
**Birth Weight (g)**^**a**^ (range)	1177 ± 342 (630–1740)	1018 ± 243 (599–1585)	ns^#^
**Gender (n; male/female)**	13/12	16/10	
**Ethnicity (%)**			
- Caucasian	52	42	
- African-American	20	29	
- Asian	4		ns^¶^
- Hispanic (white)	24	29	
**Education (%)**			
- Secondary school	17	29	
- University	57	50	ns^¶^
- Post-Graduate	26	21	
**Income (%)**			
< $50,000	43	42	
- $50,000–$99,999	30	37	ns^¶^
- ≥ $100,000	26	21	

^a^Mean ± SD

^#^ Independent t-test: significance at *p* ≤ 0.05

^¶^ Chi Square analysis: significance at *p* ≤ 0.05

^¥^ WinPEPI (Compare2)

As maternal scores with Defensive Responding ≤24 may not be representative of their true sentiments, data analyses were conducted separately for “Non-Defensive (ND; scores >24)”, and “Defensive (D; scores ≤ 24)” mothers (Fig. 1). No significant difference in PSI subscales was noted within ND and D mothers at 1- vs. 12-month CA, with the exception of the Child Mood subscale that was significantly greater in the ND group (MO; *p* = 0.03; Fig. 1). Due to the similarity in responses at 1- vs. 12-month CA visits within ND and D group of mothers, scores of the PSI subscales at 1- and 12-month CA were *averaged* (Fig. 1, solid line) to provide a “*profile*” to compare between these two groups. The average scores for the majority of subscales were significantly lower in the D vs. ND mothers (*p* < 0.001) with the exception of DI (Distractibility/hyperactivity in the Child Domain) and LS (Life Stress in the Parent Domain) scores.

Stress scores in the ND mothers fell within the 15th to 80th %ile of the general population range of Abadin’s normative population. D mothers’ %ile scores for the majority of the PSI scores (10/17) were below the 15th %ile (Fig. 1). In the Marlowe-Crowne Social Desirability test, ND mothers demonstrated lower scores (19 ± 38; mean ± SD) than their D counterparts (24 ± 15; *p* = 0.05), suggesting that ND mothers were more truthful in their responses than their D counterparts.

**Fig. 2:**
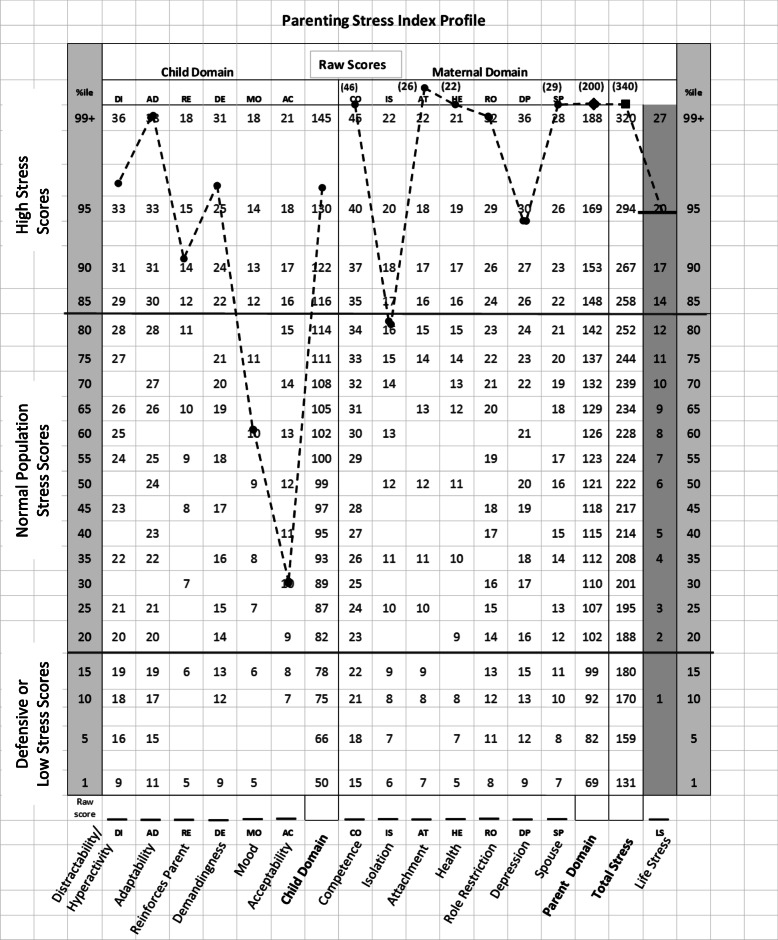
Profile of a ND mother with high stress scores (> 85%ile) for the majority of subscale levels at 1-month CA visit. Raw scores > 99%ile noted above corresponding subscales

Fig. 2 shows the PSI scores of a mother (36 years old) for one of her infants at the 1-month CA visit. She delivered triplets at 32 weeks gestation following Artificial Reproductive Technology (ART). With a Defensive Responding score > 24, she was included in the ND group. Her infants were discharged from the NICU on their 38th week postmenstrual age (PMA) and she returned to work at 7 weeks postpartum. She was at the highest level for Education and Income. But for three of her subscales, MO, AC in the Child Domain and IS in the Parent Domain, her scores fell above the high score range of 85%ile of Abidin’s normative population. The actual scores for CO, AT, HE, SP and PD in the Parent Domain, being greater than the 99%tile of Abidin’s range at 1-month CA, are noted above each corresponding subscale. This mother was lost to subsequent follow-up assessments.

## Discussion

The primary aim of this study was to gain a better understanding of the stress *perceived* by mothers during their first year when reunited with their infants post-NICU discharge. As mothers did not have any concern over their infants’ growth and development during their follow-up visits at the Texas Children’s Hospital GCRC, we reasoned that the demands imposed on them may be comparable to those who lived in the general community of Abidin’s normative population. As such, the PSI was our instrument of choice because it offered the possibility to compare how different these mothers’ *perceived* stress at their infants’ term equivalence of 1- and 12-month CA could be from that of a normative population of mothers from the community.

Our observations may be summarized as follow. First, there was no significant difference in parental stress scores between the 2 groups of mothers monitored at 1- and 12-month CA, whether they fell in the Non Defensive (ND) or Defensive (D) group. Second, ND mothers demonstrated significantly greater stress scores than their D counterparts for the majority of the subscales. Third, the average maternal Life Stress scores, i.e., stress experienced *outside* of the parent-child interactions were similar between ND and D mothers, suggesting that the differences in scores observed within the Child and Mother domains were specifically associated with the interactions they had with their infant. Fourth, the *profiles* in the Child and Parent Domains for both ND and D mothers demonstrated similar peaks and troughs, e.g., child Mood, Health, Spouse, at 1- and 12-month CA with the exception of Child Mood for the ND mothers, suggesting that these particular concerns may not be associated with individual maternal characteristics, but rather with the responsibility they had towards their infant and spouse.

With the observation that maternal *perceived* stress did not subside during their infant’s first year at home, it is proposed that early identification of “at-risk” mother-infant dyads is advantageous as the well-being of the dyad is as important as that of the infant. The PSI could be readily administered at their infants’ 1- and 12-month CA visits normally scheduled with their pediatrician. It is expected that by the 1-month CA visit, both partners of the dyad would have had time to establish a daily routine.

The importance of such evaluation is demonstrated by the subject presented in Fig. 2. She is an example of how early awareness of her PSI scores upon reunification with her infants at home could have warned care providers of her struggles. This mother of triplets had 14 out of 17 PSI subscales > 85%ile at her 1-month CA visit. Concerns over her well-being were warranted as her Total Stress, Life Stress and Health were 340, 20, and 22, respectively. PSI recommends referral for professional assistance when these 3 scores are in the 250 range, ≥ 17, and > 16, respectively. As recommended in clinical research studies for outliers, she was provided referrals to appropriate services for further assistance by our clinical psychological associate (MRT). Unfortunately, as our study was focused on the mother-infant dyad rather than the individual partners of the dyad, she was lost to follow-up after her 1-month CA visit and it is unclear whether she received any subsequent support. In our view, such unfortunate incidence results from the fact that mothers of preterm infants are not patients per se*,* unlike their infants. Unless they had previous medical issues, they do not fall under the care of any healthcare provider following their regular postpartum follow-up appointment with their obstetrician.

Identification of elevated stress scores in any of the PSI subscales provides an objective framework that could facilitate early targeted guidance and social support to help mothers develop more constructive parenting routines. As the PSI can be re-administered over time, the efficacy of such approaches could be readily verified.

As parenting stress during the first 3 years of life is critical to a child’s emotional and behavioral development as well as for the appropriate development of a parent-child relationship (Parental Stress Index, 3rd Ed, p,1 [26, 39];), the earlier the identification of mother-child imbalanced exchanges, the greater the potential for” disentangling […] mother-infant dyadic processes” [40]. Unfortunately, there is currently no routine screening process, to our knowledge, that addresses such issue. As mothers are not considered patients per se*,* caregivers’ emphasis rightfully is placed on the child’s welfare through regular pediatric visits rather than on both the child and the “healthiness” of mother-child intimate exchanges.

Mother-child synchrony is a co-regulatory process involving both physiologic and behavioral elements. During “balanced” positive interplays, synchronized coupling of mother and infant’s physiologic functions are observed, e.g., cardiac and diurnal neuro-endocrine rhythms, specific central nervous system networks [16, 41, 42].

As a mother’s responsibility is first and foremost aimed at meeting her infant’s needs for proper growth and development, she may be considered the more “pro-active” partner of the dyad. The literature describes a cooperative balanced interaction in which mother and infant continually “re-adjust” their actions towards one another’s needs and a controlling one in which mothers take over the direction of the exchanges [14, 17, 23]. Cooperative synchronous and healthy bidirectional exchanges will likely reduce their respective levels of emotional and behavioral conflicts, while strengthening their bond [43–45]. In contrast, under a controlling pattern, mothers who downplay their child’s needs may unduly increase mother-child conflicts leading to asynchronous interactions and raising both partners’ stress levels as infant negative reactivity will likely arise [14, 23, 46–49].

Although clinical and animal studies conducted on maternal stress have focused more heavily on lactation and its maternal psychological/emotional reactions [13, 24, 29, 50–55], less emphasis has been placed on how the nature of the infant developmental/behavioral feedback impact positively or negatively on maternal functions [14]. The PSI would be a useful tool for such purpose as incorporating maternal *perception* on how particular infant comportment/behaviors are would further assist health professional ‘tease out’ infant and parental factors that have greater detrimental impact on their continual exchanges [56, 57]. Once home, the interactions of a mother and infant may be exacerbated not only by their respective personal attributes, but also by the multitude of dynamics outside their control. Under such environment, it becomes difficult to identify the more leading ‘culprit(s)’ that threaten the integrity of the dyad. With a mother’s PSI profile reflecting her *perceived* reactions to her infant and her own PSI subscales, caregivers could gain a head start when assisting mothers, an approach that could promote positive parenting [58]. Although the PSI does not offer objective temperamental/behavioral measures and may not be well received by researchers, “clinicians know that it does not matter from a parent’s perspective if a child is active in an *absolute* sense, but it does matter whether the level of activity is excessive and disruptive to the parent” (PSI-3, PAR Inc., p.2). Maternal self-reporting of the sources of stress experienced, although subjective, determine their responses towards their infant’s behaviors. As such, we deemed the use of this instrument to be a realistic “first step” in understanding the unique clinical population represented by the mother-preterm infant dyad.

As participants’ stress levels for most of the PSI subscales were not different between groups of mothers at 1- and 12-month CA, it is suggested that maternal stress levels did not subside despite presumed maternal adjustment to motherhood. As infants’ maturation leads to ever changing behaviors and demands, one may presume that maternal adjustments are a never-ending process. The observation that the PSI profiles of our ND participants were within the range of Abidin’s normative population also suggests that, irrespective of the types of stressors implicated, maternal level of parenting stress at home was within the range of the general population. Gray et al. [59] in a study using the PSI short form observed that maternal Total Stress score of preterm infant (≤30 wks GA) tested at 12-month CA were significantly higher than counterparts born term at 12-month of age, albeit both groups of mothers were within the 15–80%ile of Abidin normative population. It is noteworthy to mention that a number of their preterm recruits experienced varied developmental impairments, e.g., cerebral palsy and Griffiths Mental Development issues, whereas our cohort did not have any medical concern as reported by their mothers.

There may be several reasons why D mothers showed lower intensity scores than their ND counterparts. Being more sensitive to social recognition, they may have downplayed their stress. This is supported the observation that their Marlowe-Crowne Social Desirability scores were significantly more elevated in the D vs. ND mothers. It is also conceivable that the lower stress score of D vs. ND mothers resulted from an increased protection of their infant, a behavior observed in animals who demonstrate decreased circulating levels of stress hormone following the birth of their young [50, 51]. From a positive perspective, it is plausible that, as mentioned earlier, their scores were truthful because of their high competency caring for their infant and/or their supportive family/social environment. However, the observation that maternal perception of “Life Stress”, i.e., scores pertaining specifically to stressors perceived by mothers that are *outside the parent-child relationship*, eg., spouse, social activities, work etc..., was similar between ND and D mothers would support the notion that the Defensive Responding approach of D mothers pertained to interactions with their infant rather than their high competency in caring for their infant or supportive environment.

As groups of population share common attributes/characteristics, the PSI offers an additional advantage. It has been used to obtain an ‘average’ characteristic profile of specific clinical groups, e.g., children with sensory, speech, mental, or motor impairments, cerebral palsy, Down’s syndrome, autism, maternal substance abuse, etc. (PSI-3, PAR Inc. [60];. In a similar manner, this is the first study, to our knowledge, that provides a PSI *profile* of mothers reunited with their infants following their NICU discharge. It is advanced that this profile may assist caregivers better understand and assist the growing clinical population of mothers of premature infants.

## Conclusion

In summary, further validation of the PSI for preterm mother is warranted to substantiate its potential benefits as an *early screening* instrument for the early identification of at-risk mother-preterm infant dyads. The different PSI subscales within the Child and Parental domains would facilitate the development of individualized interventions focused on the more specific needs of *individual* mothers. It is advocated that properly targeted interventions could assist caregivers help mothers build a positive mutualistic parenting routine for themselves and their babies.

## Data Availability

All data generated or analyzed during this study are included in this published article [and its supplementary information files].
